# Comparison of Traditional and Advanced Resistance Training Paradigms on Muscle Hypertrophy in Trained Individuals: A Systematic Review and Meta-Analysis

**DOI:** 10.1155/2023/9507977

**Published:** 2023-07-18

**Authors:** Pedro A. B. Fonseca, Bernardo N. Ide, Dustin J. Oranchuk, Moacir Marocolo, Mário A. M. Simim, Michael D. Roberts, Gustavo R. Mota

**Affiliations:** ^1^Exercise Science, Health and Human Performance Research Group, Department of Sport Sciences, Institute of Health Sciences, Federal University of Triângulo Mineiro, Uberaba, MG, Brazil; ^2^Sports Performance Research Institute New Zealand, Auckland University of Technology, Auckland, New Zealand; ^3^Acumen Health, Calgary, AB, Canada; ^4^Department of Physiology, Federal University of Juiz de Fora, Juiz de Fora, MG, Brazil; ^5^Physical Education and Adapted Sports Research Group, Institute of Physical Education and Sports, Federal University of Ceará, Fortaleza, Brazil; ^6^School of Kinesiology, Auburn University, Auburn, AL, USA

## Abstract

Trained individuals may require variations in training stimuli and advanced resistance training paradigms (ADV) to increase skeletal muscle hypertrophy. However, no meta-analysis has examined how ADV versus traditional (TRAD) approaches may differentially affect hypertrophic outcomes in trained populations. The aim of this review was to determine whether the skeletal muscle hypertrophy responses induced by TRAD differed from ADV in resistance-trained individuals. Furthermore, we sought to examine potential effects of dietary factors, participants' training status, and training loads. We searched for peer-reviewed, randomized controlled trials (published in English) conducted in healthy resistance-trained adults performing a period of TRAD and ADV with pre-to-post measurement(s) of muscle hypertrophy in PubMed, Web of Science, SPORTDiscus, and MEDLINE databases up to October 2022. A formal meta-analysis was conducted in Revman5, and risk of bias was assessed by ROB2. Ten studies met the inclusion criteria. Results indicated no difference between ADV and TRAD for muscle thickness (SMD = 0.05, 95% CI: −0.20 0.29, *p* = 0.70), lean mass (SMD = −0.01, 95% CI: −0.26 0.23, *p* = 0.92), muscle cross-sectional area (SMD = −0.07, 95% CI: −0.36 0.22, *p* = 0.64), or all measurements analyzed together (SMD = −0.00, 95% CI: −0.15 0.14, *p* = 0.95). No heterogeneity or inconsistencies were observed; however, unclear risk of bias was present in most of the studies. Short-term ADV does not induce superior skeletal muscle hypertrophy responses when compared with TRAD in trained individuals. This review was not previously registered.

## 1. Introduction

The ability of the skeletal muscle to generate strength and power is primarily dictated by neural drive (i.e., motor unit firing rate and frequency) and the quantity of muscle contractile and structural proteins [1, 2]. Under appropriate nutrient provision, resistance training (RT) optimizes the accretion of contractile and structural proteins and promotes skeletal muscle hypertrophy [3–5]. Despite recent investigations challenging the hypothesis that RT-induced increases in muscle size meaningfully contribute to increases in muscle strength [6, 7], athletes often seek to maximize a hypertrophic response to training with the general acceptance that this translates into performance gains [5].

The American College of Sports Medicine suggests that moderate loading (70–85% of one-repetition maximum (1RM)) with 8–12 repetitions per set, for 1–3 sets per exercise, is effective for facilitating muscle hypertrophy in novice (untrained individuals with no RT experience, or who have not trained for several years) and intermediate trainees [8]. However, for individuals that possess an advanced training status, a loading range of 70–100% of 1RM with 1–12 repetitions per set for 3–6 sets per exercise in a periodized manner is recommended such that the majority of training is devoted to 6–12RM training and less training is devoted to 1–6RM loading [8].

The above recommendations are related to the fact that, while untrained individuals can develop strength using any reasonable RT program [9], the potential for further functional and morphological improvements diminishes as an individual becomes more well trained. In this regard, a window of adaptation in trained individuals may exist [1, 10], resulting in slower rates of strength and hypertrophy increases than in untrained individuals [9, 10]. To avoid a “plateau” in skeletal muscle adaptation, reputable strength and conditioning guidelines advise that trained individuals may require higher variations in training stimuli, more sophisticated planning strategies, and longer training periods to achieve changes in strength and hypertrophy [11, 12].

These and associated recommendations for novice and advanced training statuses are often denoted as traditional RT approaches (TRAD) [13]. Conversely, advanced RT paradigms (ADV), or specialized training techniques advocated to optimize muscle growth, include the utilization of drop-sets, forced repetitions, rest-pause repetitions, super slow repetitions, pyramid sets, pre-exhaustive sets, supersets, accentuated eccentric overload, and German volume training [13–15]. Some advanced RT paradigms have been investigated and compared to TRAD regarding the potentiation of muscle hypertrophy in resistance-trained individuals [13, 15]. However, a recent narrative review [15] concluded that the currently available evidence could not determine whether ADV variations can optimize muscle strength and mass gains compared to TRAD.

Several studies [13, 16, 17] have examined RT adaptations in well-trained participants following short-term RT interventions (i.e., 6–12 weeks). However, no meta-analysis has examined how different RT paradigms (e.g., TRAD versus ADV) may affect hypertrophic outcomes in previously trained individuals. Therefore, this systematic review and meta-analysis sought to determine whether the skeletal muscle hypertrophy responses induced by TRAD differ from ADV in resistance-trained individuals. Based on previous literature [13, 15–17], we hypothesized that ADV and TRAD would elicit similar effects regarding muscle hypertrophy responses.

## 2. Methods

### 2.1. Eligibility Criteria

This review is in line with the current Preferred Reporting Items for Systematic Reviews and Meta-Analysis (PRISMA) checklist [18]. Population, Intervention, Comparator, Outcome, and Time (PICOT) [19] strategy was adopted (P: trained individuals, I: ADV, C: TRAD, O: hypertrophy, and T: intervention time described at least on number of sessions or weeks). Inclusion criteria for studies were as follows: (i) peer-reviewed, published in English, and available as a full-text manuscript; (ii) randomized controlled trials conducted with healthy resistance-trained adults performing a period of TRAD and ADV; (iii) measurement of skeletal muscle hypertrophy pre-to-post training change scores at the macroscopic and microscopic level with the following techniques: B-mode, panoramic, extended field of view or three-dimensional ultrasonography, dual-energy X-ray absorptiometry (DEXA), computed tomography, peripheral quantitative computed tomography, magnetic resonance imaging, muscle biopsies, and/or measurement of lean body mass change by plethysmography; (iv) RT program presented as ADV must match the description provided by previous literature (see Advanced Paradigms section); and (v) raw data (i.e., mean, standardized deviation, median, and standardized error) provided in the text, Table(s), or Figure(s). Studies observing responses to low-load blood flow restriction, non-isoinertial RT (e.g., flywheel, isokinetic, and pneumatic devices), creatine, protein or other supplements, anti-inflammatory drugs, or the influence of training frequency were not included.  Section 2.2 describes ADV identified and used for classifying and including the studies reviewed. If there was any divergence in the selection of studies between the two reviewers, a third reviewer was included for the final decision.

### 2.2. Advanced Resistance Training Paradigm (ADV) Description

Advanced paradigms consist of pre-defined RT protocols based on the configuration of RT variables (i.e., load, number of repetitions and sets, movement velocity, rest intervals between sets, exercises or repetitions, or exercise order, among others).

#### 2.2.1. Accentuated Eccentric

Accentuated eccentric or eccentric overload aims to provide a greater load in the eccentric phase of the movement [15, 16]. The concentric phase is performed with a regular load (e.g., 70% of 1RM) whereby the load is adjusted for the eccentric phase (usually above the concentric 1RM, e.g., 110–120% of 1RM), which requires external assistance [16].

#### 2.2.2. Drop-Sets

Drop-sets involve reducing the load (e.g., 20%) to perform additional repetitions after achieving failure in a set [14, 20]. The process can be repeated on the same set, and a minimal rest interval is allowed between load reductions [14, 20].

#### 2.2.3. Forced Repetitions

After achieving concentric failure during a set, proper assistance (i.e., by the coach or partner) is provided to the lifter to perform additional repetitions [14].

#### 2.2.4. German Volume Training

German volume training is characterized by the performance of 10 sets of 10 repetitions in no more than two exercises with a load of approximately 60% of 1RM [15, 21].

#### 2.2.5. Paired Sets

Paired sets, supersets, or bi-sets are described as the combination of two exercises executed in sequence without rest [14]. Supersets are considered a specific agonist-antagonist combination of exercises [14], and a variation of a bi-set with three exercises is also known as tri-set [15].

#### 2.2.6. Pre-Exhaustion

A single-joint exercise set is performed until failure immediately before a set of a multi-joint exercise of the same muscular group to induce more fatigue in a specific muscle [15, 22].

#### 2.2.7. Pyramid

The pyramid system consists of a configuration of sets leading to a progressive increase (i.e., crescent pyramid) or decrease (i.e., decrescent pyramid) in the load for each set performed [13, 15]. The number of repetitions performed follows an inverse relationship pattern for each configuration [13, 15].

#### 2.2.8. Rest-Pause

An overestimated number of repetitions are fixed to a given load. When failure is reached, a short rest interval (e.g., 20 seconds) is taken before subsequent repetitions are performed until failure is achieved again [15, 23].

#### 2.2.9. Super Slow

Super slow training is characterized by using a very slow movement velocity for each repetition (e.g., 10 seconds to concentric and 4 seconds to eccentric) [15, 24].

### 2.3. Information Sources

The search was conducted in PubMed, Web of Science, Scopus, SPORTDiscus with Full Text, and MEDLINE Complete databases using a specific syntax described in Section 2.4. The initial search was conducted in January 2022. A final search was conducted on October 17, 2022. References lists of included studies were also examined for potential studies not found on initial search.

### 2.4. Search Strategy

Based on the PICOT strategy, we developed a specific syntax to conduct the search on all databases. Searches were conducted without filters or limits (not advanced search) using the following terms: (“resistance train∗” OR “strength train∗” OR “weight train∗”) AND (“accentuated eccentric” OR “drop-set” OR “super-slow” OR “pyramid∗” OR “pre-exhaustion” OR “eccentric overload” OR “rest-pause” OR “German volume training” OR “forced repetition∗” OR superset OR “bi-set” OR “tri-set”) AND (hypertrophy OR “muscle mass” OR “fiber cross-sectional area” OR “muscle thickness” OR “muscle volume”) AND (session∗ OR week∗).

### 2.5. Selection Process

The studies founded on initial search were imported into the software Rayyan online for systematic reviews [25] to find duplicates and perform the screening record according to the inclusion criteria by two different reviewers (PF and BI). Rayyan software allows both reviewers to conduct the entire process in blind mode. Once both reviewers have completed their screening, the blind mode is turned off. If any discrepancies arise, they are resolved by consensus with a third reviewer (GM).

### 2.6. Data Collection Process

One author (PF) extracted data from the included studies, and a second author (BI) double-checked the data. No automatic tools were used. Disagreements were resolved through personal communication between the authors.

### 2.7. Data Items

Skeletal muscle hypertrophy was extracted from each study at the pre- and post-intervention time points for all measurements reported (i.e., types of muscle hypertrophy assessment or local of measurement). Only variables related to skeletal muscle hypertrophy were considered for calculating standardized mean differences (SMDs).

### 2.8. Study Risk of Bias Assessment

Following recommendations for randomized controlled trials, the risk of bias was assessed by the scale Risk of Bias-2 scale of Cochrane [26, 27] by two reviewers (PF and BI). The domains assessed were the randomization process (A), deviations from the intended interventions (B), missing outcome data (C), measurement of the outcome (D), and selection of the reported result (E). The overall risk of bias was determined according to each study's higher risk domain (F) presented. The assessment was done by answering the pre-specified questions about the adequacy of each study. The analysis was conducted according to recommendations using software provided by Cochrane. According to the pre-specified questions, the studies were classified in each domain as low, unclear, and high risk of bias. The overall risk of bias was determined by the higher risk attributed to any domain.

### 2.9. Effect Measures

For all types of hypertrophy measurements (i.e., pre to post differences), an analysis of SMD between ADV and TRAD was conducted. Results were considered trivial, small, moderate, and high with the following values: <0.2, ≥0.2 and < 0.5, ≥0.5 and < 0.8, and ≥0.8.

### 2.10. Synthesis Methods

All studies that presented hypertrophy measures were analyzed independently of type of measurement reported. However, a sub-analysis for each type of measurement was conducted (see Results section). The Review Manager software Version 5.4 (the Cochrane Collaboration, 2020) was used for data entry, statistical analysis, and plotting the figures [27]. No additional preparation of data was required. The level of between-study heterogeneity was assessed using the chi-square (*χ*^*2*^) test and I-square (*I*^*2*^) statistic [28]. *I*^*2*^ outcomes of 25, 50, and 75% correspond to low, moderate, and high heterogeneity [29], with a value of 0% indicating no heterogeneity, and above 75% were rated as heterogeneous. A meta-analysis with SMD, the degree(s) of freedom (d*f*), and the 95% confidence interval (CI) was reported. The model of effect analysis was chosen according to the heterogeneity of the studies, fixed-effect model for no heterogeneity (i.e., *I*^*2*^ of 0%) or random-effect model to any presence of heterogeneity (i.e., *I*^*2*^ > 0%). Differences at the level of *p* < 0.05 were considered statistically significant. Additionally, to evaluate the robustness of the results, we performed a sensitivity analysis using the exclusion of specific studies method.

### 2.11. Reporting Bias Assessment

The reporting bias of each study was accessed through the* E* scale of ROB2, and an analysis of possible publication bias was also conducted with visual analysis of the funnel plot, Egger's regression, and Begg and Mazumdar rank correlation tests. The tests were conducted on Jamovi software [30].

### 2.12. Certainty Assessment

In this systematic review, the Grading of Recommendations Assessment, Development, and Evaluation (GRADE) approach was used to assess the certainty of the evidence [31]. The certainty of the evidence was assessed for hypertrophy outcome using the GRADE framework.

## 3. Results

**Figure 1:**
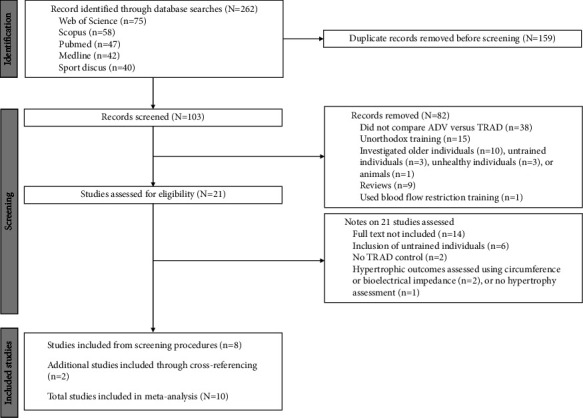
Flowchart illustrating the distinct phases of the search and selection strategy.

A total of 262 records were found from Web of Science (*n* = 75), Scopus (*n* = 58), PubMed (*n* = 47), MEDLINE Complete (EBSCO, *n* = 42), and SPORTDiscus with Full Text (EBSCO, *n* = 40). After removing duplicates, 103 records remained. According to inclusion criteria, 22 studies were considered possibly eligible. After full-text assessments, 14 studies were excluded. In addition to the eight studies, two additional studies were included after consulting the articles' reference lists. This led to 10 studies being included in the final analysis.  Figure 1 shows the flowchart diagram of the study screening process.

### 3.1. General Description of the Studies

The description of studies regarding the RT programs that investigated muscle hypertrophy outcomes is presented in Table 1.

Seven different types of ADV were identified including German volume training in two studies [21, 32], crescent pyramid in one study [13], drop-sets and heavy drop-sets in three studies [13, 17, 33], eccentric overload/accentuated eccentric in three studies [16, 34, 35], pre-exhaustion in one study [36], super slow in one study [34], and rest-pause in two studies [17, 37].

Different types of hypertrophy assessments were identified, including B-mode ultrasonography MT with 14 comparisons, lean body mass via DEXA or air displacement plethysmography with 13 comparisons (Walker et al. [35] also performed lean body mass analysis; however, data were incomplete and were not included in the calculation of SMD), and anatomical cross-sectional area (ACSA) via B-mode ultrasonography or magnetic resonance imaging with seven comparisons. Five studies [13, 17, 21, 35, 37] reported significant hypertrophy changes after interventions, and two studies [21, 35] reported significant changes in MT but not in lean mass for both TRAD and ADV.

### 3.2. Comparison of TRAD and ADV on Muscle Hypertrophy

**Figure 2:**
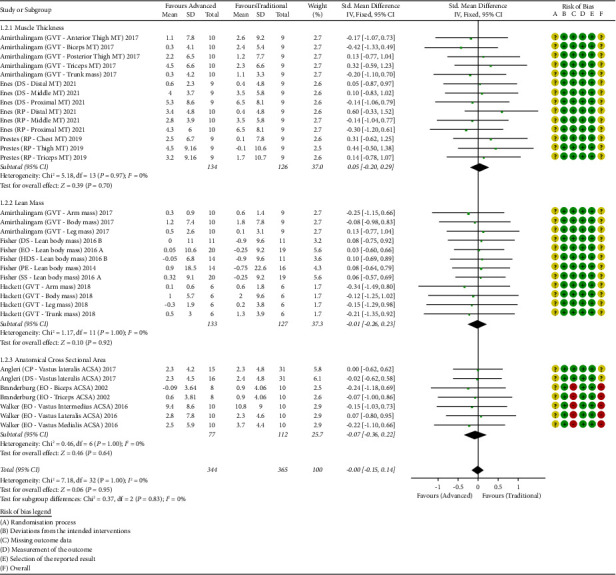
Forest plot of the analyses and risk of bias. SD: standardized deviation; GTV: German volume training; MT: muscle thickness; DS: drop-set; RP: rest-pause; EO: eccentric overload; HDS: heavy DS; PE: pre-exhaustion; SS: super slow; ACSA: anatomical cross-sectional area; CP: crescent pyramid; risk of bias legend: A, randomization process; B, deviations from the intended interventions; C, missing outcome data; D, measurement of the outcome; E, selection of the reported result; F, overall risk of bias.

Some studies contained multiple groups (i.e., more than one ADV group, resulting in 24 groups) or multiple hypertrophy analyses (i.e., lean body mass, ACSA, and muscle thickness (MT) in different locations). Therefore, 33 comparisons were considered for the meta-analysis (see Figure 2). Control groups that have trained with their own previous RT routines (i.e., outside the laboratory) were not considered. One study [36] compared two protocols with pre-exhaustion (called TRAD and control, see Table 2). Only TRAD was included in the analysis as the control group altered the sequence in the study [36].

The forest plot of all included comparisons is presented in Figure 2.

#### 3.2.1. Muscle Thickness Changes

Separate analysis of MT indicated no heterogeneity between studies (*p* = 0.97, *I*^*2*^ = 0%). Considering a fixed-effect model, the analysis of SMD showed no difference between ADV and TRAD when MT was used as hypertrophy assessment (SMD = 0.05 CI: [−0.20 0.29]).

#### 3.2.2. Lean Body Mass Changes

Separate analysis of lean body mass changes indicated no heterogeneity between studies (*p* = 1.00, *I*^*2*^ = 0%). Considering a fixed-effect model, the analysis of SMD showed no difference between ADV and TRAD when lean body mass was used as hypertrophy assessment (SMD = −0.01 CI: [−0.26 0.23]).

#### 3.2.3. Anatomical Cross-Sectional Area Changes

Separate analysis of ACSA indicated no heterogeneity between studies (*p* = 1.00, *I*^*2*^ = 0%). Considering a fixed-effect model, the analysis of SMD showed no difference between ADV and TRAD when CSA was used as hypertrophy assessment (SMD = −0.07 CI: [−0.36 0.22]).

#### 3.2.4. All Muscle Hypertrophy Assessments

Analysis of all hypertrophy measurements together indicated no heterogeneity between studies (*p* = 1.00, *I*^*2*^ = 0%). Considering a fixed-effect model, the analysis of SMD showed no difference between ADV and TRAD (SMD = −0.00 CI: [−0.15 0.14]).

### 3.3. Dietary Controls

Table 3 shows a summary of the dietary control reported in each study.

Only six studies presented some type of dietary control [13, 17, 21, 32, 35, 37]. Four studies instructed their participants how to proceed with their nutritional intake habits during the period of the study [13, 17, 21, 32], four studies provided a standardized protein supplementation after exercise [13, 21, 32, 35], and two studies calculated nutritional intakes from dietary records [17, 37]. One of these studies [37] did not report data related to dietary controls.

### 3.4. Participants' Training Statuses

Table 4 shows participants' characteristics, RT experience, training status reported in the study, and training status according to the scale suggested by Rhea [38].

Four studies [13, 17, 21, 35] reported time of experience in RT of participants, and the other six reported the minimum of time experience required for a participant to be eligible for the study [16, 32–34, 36, 37]. According to Rhea's classification of training status [38], six studies [21, 32–36] included untrained subjects, four studies [13, 16, 17, 37] included recreationally trained subjects, and no study included only highly trained individuals. However, one study reported individuals that varied from untrained to recreationally trained [32], two studies from untrained to highly trained [21, 35], and one study from recreationally trained to highly trained [13].

### 3.5. Quantification of Training Loads

The quantification of training loads described during training interventions and the participants' previous training experience are presented in Table 2.

None of the studies reported complete data about the quantification of training loads. Two studies [13, 17] reported the total volume load. Angleri et al. [13] reported a total of ∼150 tons executed in TRAD, drop-set, and crescent pyramid, while Enes et al. [17] reported 412263 ± 50764 kg for drop-set, 440363 ± 45953 kg for rest-pause, and 405428 ± 45748 kg for TRAD. One study [32] reported the average volume load of the sessions, but for less than a fifth of the exercises performed. The mean volume load of sessions for German volume training was 4879 ± 773 kg, and it was 24491 ± 4180 kg for the bench press and leg press, respectively [32]. The mean volume load of sessions for TRAD was 2407 ± 483 kg and 13498 ± 2712 kg for the bench press and leg press, respectively [32]. Additionally, one study [21] reported the volume load for two sessions only (initial and final) and three exercises (bench press, cable pull-down, and leg press) only. Volume load of these three exercises on the initial session was 4583 ± 852 kg, 3962 ± 712 kg, and 20901 ± 9942 kg for GTV and 1845 ± 700 kg, 1596 ± 408 kg, and 10117 ± 2636 kg for TRAD, respectively, for bench press, cable pull-down, and leg press. Volume load of these three exercises on the final session was 5078 ± 775 kg, 3862 ± 689 kg, and 24883 ± 3424 kg for German volume training and 2329 ± 766 kg, 1826 ± 444 kg, and 12941 ± 3051 kg for TRAD, respectively, for bench press, cable pull-down, and leg press.

We attempted to examine the RT programs performed by the participants before their engagement in the included studies. However, none of the studies reported the RT program performed by the participants before their engagement in the study. Only one study [13] estimated the previous volume loads performed by the participants two weeks before engagement in the study; however, the data were unavailable. One study reported the usual ranges of sets and repetitions performed by the participants [37]. One study [13] reported previous experience with exercises used in the intervention.

### 3.6. Risk of Bias Analysis

**Figure 3:**
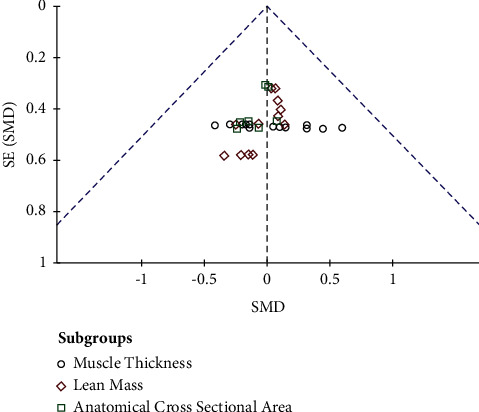
Funnel plot of included studies. SE: standardized error for SMD; SMD: standardized mean difference.

Analysis of the risk of bias revealed that only two studies [16, 35] had a high risk of bias due to unequal dropouts of the ADV group (domain C of Risk of Bias-2 scale), both of which investigated eccentric overload. All studies presented a lack of information in domain A (which does not inform if the allocation was concealed), resulting in an unclear risk (F) for the remaining eight studies. However, most studies had a low risk in domains B–E. A summary of the risk of bias analysis is illustrated in Figure 2, while the funnel plot of all included comparisons is presented in Figure 3.

Visual inspection of the funnel plot reveals that the results were unlikely to be influenced by publication risk bias [39]. Likewise, Egger's regression and Begg and Mazumdar rank correlation were used to evaluate publication bias. Both tests showed that there was no risk of publication bias (Egger's regression: −0.352, *p* = 0.725; Begg and Mazumdar rank correlation: −0.025, *p* = 0.844). No heterogeneity was found, and the analysis of sensitivity revealed that exclusion of any study did not alter the results of the meta-analysis.

### 3.7. Certainty Assessment

We used the GRADE framework to assess the certainty of evidence for hypertrophy outcomes. Many factors of the studies analyzed support a high level of quality evidence such as studies were randomized controlled trials, consistently similar results were obtained, the measurements used by studies are direct to the variable(s) of interest, no heterogeneity was found (i.e., *I*^*2*^ = 0%), and a low probability of publication bias was present (i.e., no significant Egger's regression or Begg and Mazumdar rank correlation tests). However, two studies were found to have a high risk of reporting bias according to ROB2 analysis. Additionally, none of the studies mentioned a concealment process during randomization, resulting in an overall unclear risk of bias. Due to these factors and the lack of nutritional control and proper reporting of training loads, we assigned a moderate level of quality of evidence for our systematic review's conclusions. Future studies in this area could leverage these findings to improve study designs in this regard.

## 4. Discussion

The aim of this meta-analysis was to determine whether the skeletal muscle hypertrophic responses induced by TRAD are different from ADV in resistance-trained individuals. Our results indicate that, regardless of skeletal muscle hypertrophy assessment (i.e., MT, lean mass, or ACSA), no significant advantage was provided by ADV versus TRAD (see Figure 2). This finding corroborates with our hypothesis and previous literature [13, 15].

### 4.1. Comparison of TRAD and ADV on Muscle Hypertrophy

Most of the included studies did not report differences in outcomes between ADV and TRAD (see Table 1). These data suggest that skeletal muscle hypertrophy may not be enhanced through 6–12 weeks of ADV in previously trained individuals. However, one study [37] reported significant increases in thigh MT differences after six weeks of the rest-pause system compared to TRAD (11% increase for rest-pause, and no increases for TRAD, see Table 1). Analysis of MT may be accurate to estimate muscle size (i.e., muscle volume assessed by magnet resonance image) when considering a single time point assessment [40]. However, when assessing chronic muscle hypertrophy changes, MT has some limitations [40] associated with muscle physiology (i.e., heterogeneous distribution of hypertrophy [41, 42]) and the geometric nature of the measure that is limited to a specific site of the muscle [40]. Moreover, despite this study presenting differences in thigh MT, no differences in the chest and arm MT were found [37].

Curiously, five of ten studies included in this review failed to observe hypertrophy in both groups (i.e., ADV and TRAD [16, 32–34, 36]). Since these studies aimed to compare hypertrophy changes induced by TRAD and ADV, failure to achieve skeletal muscle hypertrophy in both groups is a limitation. Small sample size [32], lack of dietary controls [21, 33, 34, 36], lower sensitivity of some measurements in detecting hypertrophy changes (e.g., plethysmography [33, 34, 36]), and inconsistencies in training load monitoring [16, 32–34, 36, 37] may be among the possible candidates to explain these results.

It is also notable that the studies included in our analysis compared muscle hypertrophy outcomes using different measurement tools (e.g., MT, lean mass, or ACSA, see Table 1). This is important to note given that it has been reported that disagreements among muscle imaging techniques exist [43–45]. Thus, this remains a limitation of the current meta-analysis. Notwithstanding, our sub-group analysis of MT, lean mass, and ACSA did not reveal any differences between TRAD and ADV paradigms (SMD = 0.05 [−0.20 0.29], −0.01 [−0.26 0.23], and −0.07 [−0.26 0.23], respectively, see Figure 2). This finding lends further support that 6–12 weeks of ADV does not confer additional hypertrophic benefits in previously trained individuals.

### 4.2. Limitations

This meta-analysis is not without limitations. This review was not registered a priori. Some of the inclusion criteria implied that not all recommendations for systematic reviews were followed (e.g., choosing only English peer-reviewed randomized controlled trials). A universal definition to depict participants' training status does not exist, which may have impacted some of our conclusions regarding the influence of training status on associated outcomes. We may also have overlooked studies that failed to report participants as trained subjects. Considering that training status classification and dietary strategies were divergent among the studies reviewed, our results may only apply to recreationally trained individuals (e.g., more than one year of RT experience). Moreover, the dietary strategies could have impacted the findings herein, albeit the lack of adequate dietary data across most studies precludes us from making firm conclusions in this regard. Finally, pooling the protocols into ADV versus TRAD may present an inherent limitation in certain scenarios given that some may view German volume training (for instance) as being more traditional versus other approaches discussed herein (e.g., eccentric overload/accentuated eccentric protocols).

## 5. Practical Applications

The use of ADV is usually recommended for RT trained individuals to maximize hypertrophic responses. However, the results of this meta-analysis revealed that short-term ADV does not induce superior skeletal muscle hypertrophy responses when compared with TRAD in trained individuals.

## Figures and Tables

**Table 1:** General description of the studies.

Study	ADV investigated and duration and frequency of the program	Muscle hypertrophy assessment	Main results for TRAD	Main results for ADV	Differences between protocols
Amirthalingam 2017	GVT. 6 weeks of training performed 3 days per week.	Lean mass through DEXA and MT through B-mode ultrasonography	↑3.1, ↑4.2, and ↑7.8% in total, trunk, and arm lean mass, respectively. No significant changes in MT.	↑1.9, ↑1.0, and ↑3.5% in total, trunk, and arm lean mass, respectively. No significant changes in MT.	No significant differences between GVT and TRAD

Angleri 2017	CP and DS. 10 weeks of training performed 2 days per week.	ACSA through B-mode ultrasonography	↑7.6% in ACSA	↑7.5% and ↑7.8% in ACSA induced by CP and DS, respectively	No significant differences between CP, DS, and TRAD

Branderburg 2002	EO. The training was performed 2 days per week for the first 2 weeks and then 3 days per week for the remainder of the 9-week training period.	ACSA through magnetic resonance imaging	No significant changes	No significant changes	No significant differences between EO and TRAD

Enes 2021	DS and RP. 8 weeks of training performed 2 days per week.	MT through B-mode ultrasonography	↑14.2 and ↑6.5% in the proximal thigh and middle thigh MT, respectively. No significant changes in distal thigh MT.	↑11.6 and ↑7.7% in the proximal thigh and middle thigh MT, respectively, for DS. ↑8.8 and ↑5.1% in the proximal thigh and middle thigh MT for RP. No significant changes in distal MT for both groups.	No significant differences between DS, RP, and TRAD

Fisher 2014	PE. 12 weeks of training performed 2 days per week.	Lean mass through air displacement plethysmography	No significant changes	No significant changes	No significant differences between PE and TRAD

Fisher 2016 A	SS and EO. 10 weeks of training performed 2 days per week.	Lean mass through air displacement plethysmography	No significant changes	No significant changes	No significant differences between SS, EO, and TRAD

Fisher 2016 B	DS and HDS. 12 weeks of training performed 2 days per week.	Lean mass through air displacement plethysmography	No significant changes	No significant changes	No significant differences between DS, HDS, and TRAD

Hackett 2018	GVT. 12 weeks of training performed 3 days per week.	Lean mass through DEXA	No significant changes	No significant changes	No significant differences between GVT and TRAD

Prestes 2019	RP. 6 weeks of training performed 4 days per week.	MT through B-mode ultrasonography	No significant changes	↑11% in thigh MT	↑ in thigh MT was greater for RP

Walker 2016	EO. 10 weeks of training performed 2 days per week.	MT through B-mode ultrasonography and lean mass through DEXA	↑11 and ↑16% in vastus lateralis and medialis MT, respectively. No significant changes in lean mass^*∗*^.	↑13 and ↑11% in vastus lateralis and medialis MT, respectively. No significant changes in lean mass^*∗*^.	No significant differences between EO and TRAD

The table shows resistance training programs investigated, muscle hypertrophy assessments, and main results. GVT: German volume training; MT: muscle thickness; DEXA: dual-energy X-ray absorptiometry; CP: crescent pyramid; DS: drop-set; ACSA: anatomical cross-sectional area; EO: eccentric overload; RP: rest-pause; PE: pre-exhaustion; SS: super slow; HDS: heavy DS; ^*∗*^ = data not used in calculation of standardized mean difference due to incomplete report; ↑ = increase.

**Table 2:** Resistance training programs performed by the participants before the engagement in the study, progression, and quantification of training loads.

Study	RT program performed before the engagement in the study	Exercises performed	TRAD intervention	ADV intervention	Progression of training loads	Quantification of training load
Amirthalingam 2017	Not reported	Flat bench press; lat pull-down; incline bench press; seated row; crunches; leg press; dumbbell lunges; leg extensions; leg curls; calf raises; shoulder press; upright row; triceps pushdowns; biceps curls; sit-ups with a twist	5 sets of 10 rep with 60–80% 1RM and 60−90s of rest interval (only in 6 exercises)	GVT. 10 sets of 10 rep with 60–80% 1RM and 60−90 s rest interval (only in 6 exercises).	Loads were adjusted by 5–10% once the participants were able to complete 10 repetitions on the final set of each exercise	Only the initial and final VL for 3 exercises

Angleri 2017	Participants reported training lower limbs at least 2 d.wk.^−1^ and performing leg press 45° and leg extension	Leg press; leg extension	3–5 sets of 6–12 rep with 75% 1RM and 120 s of rest. Training occurred according to pre-established VL.	VL of DS and CP were equalized with TRAD. DS: sets were performed until failure, with a drop load of ∼20% on each failure until reaching the prescribed VL. CP: 3–5 of 6–15 rep with 65–85% 1RM and 120 s rest interval.	Initial VL was defined as 120% of the VL that each participant performed in the 2 weeks before the study. The number of sets and repetitions was adjusted every time that the VL was increased (∼7% every 3 weeks).	Total VL reported for the whole RT program

Branderburg 2002	Not reported	Preacher curl; supine elbow extension	4 sets of ∼10 rep with 75% 1RM	EO. 3 sets of ∼10 rep with 75% 1RM on concentric and 110–120% 1RM on the eccentric phase.	The load was adjusted when the average number of repetitions performed per set in a training session became greater than 10	Not reported†

Enes 2021	Not reported	Barbell back squat; 45° leg press; seated knee extension; stiff-leg deadlift; seated knee flexion	4 sets of 12 rep with 70% 1RM and 120 s of rest interval	DS. 3 sets of 10 with 70% 1RM and 3 additional sets of 6 rep with 55% 1RM with 120 s of rest between sets and no rest before additional sets (only 3 of 5 exercises). RP. 3 sets of 10 with 75% 1RM and 3 additional sets of 6 rep with 75% 1RM with 120 s of rest between sets and 20 s before additional sets (only 3 of 5 exercises).	The load was adjusted by 5% in the fifth week	Total VL reported for the whole RT program

Fisher 2014	Previous experience with PE system	Chest press; leg press; pull-down; pectoral fly; leg extension; pull-over; abdominal flexion; lumbar extension	TRAD: one set of ∼12RM and 60 s of rest. Control^*∗*^: one set of ∼12RM and 60 s of rest.	PE: one set of ∼12RM and 60 s of rest between sets and 5 s between isolated and compound exercises	Once participants were able to perform more than 12 repetitions before achieving failure, the load was adjusted by 5%	Not reported

Fisher 2016 A	Participants reported having done single-set training until failure for multiple exercises including most major muscle groups 2 d.wk.^−1^	Leg extension, leg curl; leg press; overhead press; chest press; pec-fly; pull-over; pull-down	One set of 8–12 reps with 75% 1RM with 120 s of rest interval between exercises and cadence of 6 s per rep	EO: one set of 8 reps with 105% 1RM with 60 s of rest interval between exercises and cadence of 10 s per rep (only one session; the other was realized identical to TRAD). SS: one set of 6 reps with 75% 1RM with 60 s of rest interval between exercises and cadence of 12 s per rep.	Once participants were able to perform more than desired repetitions, the load was adjusted by 5%	Not reported

Fisher 2016 B	Participants reported having done single-set training until failure for multiple exercises including most major muscle groups 2 d.wk.^−1^	Chest press; leg press; pull-down; overhead press; adductor; abductor; abdominal flexion; lumbar extension; pec-fly; pullover; leg extension; dips; biceps curl; seated calf raise; leg curl; core torso rotation	One set of 8−12RM	DS: one set of 8−12RM with additional set with reduction of ∼30% on load (only 3 exercises; 12 exercises of 15 were realized identical to TRAD). HDS: one set of ∼4RM with an additional set with two reductions of ∼20% on load (only 3 exercises; 12 exercises of 15 were realized identical to TRAD).	Once participants were able to perform more than 12 repetitions before achieving failure, the load was increased by 5% (only reported for TRAD)	Not reported

Hackett 2018	Participants reported training at least 3 d.wk.^−1^	Flat bench press; lat-pulldown; incline bench press; seated row; crunches; shoulder press; upright row; triceps pushdowns; biceps curl; sit-ups with a twist	5 sets of 10 reps with 60–80% 1RM and 60–90 s of rest interval (only in 6 exercises)	GVT: 10 sets of 10 reps with 60–80% 1RM and 60−90 s of rest interval (only in 6 exercises)	When participants were able to complete >10 repetitions on the final set, the load was increased by approximately 5–10%	Average VL for only 2 exercises (a total of 15 exercises were utilized)

Prestes 2019	The subjects were accustomed to training 3–5 days per week with split-body training routines and 3-4 sets of 8−12RM per exercise with the objective of muscle hypertrophy	Barbell bench press; dumbbell incline press; cable cross; military press; lateral raise; triceps pulley; barbell triceps extension; squat; 45° leg press; leg curl; front lat pull-down; seated row; dumbbell lateral row; standing barbell elbow curl; preacher curl	3 sets of 6 rep with 80% 1RM and 120−180 s of rest interval	RP: one set of 18 rep with 80% 1RM (performed with intra-set rests of 20 s) and 120 s of rest between exercises	No progression or adjustments were reported	Not reported

Walker 2016	Not reported	Bilateral leg press; unilateral knee extension; unilateral knee flexion	3 sets of 6RM or 10RM with 120−180 s of rest interval	EO: 3 sets of 6RM or 10RM with +40% of the load in eccentric phase and 120−180 s of rest interval	The load was adjusted to provide muscle failure in at least one of three sets	Not reported

Rep: repetitions; 1RM: one-repetition maximum test; GVT: German volume training; VL: volume load; DS: drop-set; CP: crescent pyramid; TRAD: traditional resistance training; EO: eccentric overload; RP: rest-pause; RM: repetition maximum (performed until failure); PE: pre-exhaustion; SS: super slow; HDS: heavy DS; ^*∗*^ = groups were not considered in the calculation of standardized mean difference. †= programmed volume load (calculated as sets* × *repetitions* × *percentage of 1RM) was reported to be equal between protocols.

**Table 3:** Dietary control employed in the studies.

Study	Nutritional intake record	Nutritional plan	Post-trainingstandardized supplementation
Amirthalingam 2017	The dietary intake was obtained via a 3-day food diary before and after the experimental training period	Participants were encouraged to increase their caloric intake by 1000–2000 kJ above their estimated daily energy requirements	Whey protein (30.9 g of protein, 0.2 g of fat, and 0.9 g of carbohydrate) 30 min after each training session
Angleri 2017	Not performed	Participants were advised to have a light meal 2 h before each testing session and to maintain their eating habits	30 g of whey protein after each training session
Branderburg 2002	Not performed	Not prescribed	Not prescribed
Enes 2021	Participants completed a 3-day non-consecutive dietary intake record before the intervention, at the mid-point, and conclusion of the study period. No difference in dietary intake was found between the groups.	Participants were instructed to have a meal two hours before each training session and to maintain their habitual dietary intake	Not prescribed
Fisher 2014	Not performed	Not prescribed	Not prescribed
Fisher 2016 A	Not performed	Not prescribed	Not prescribed
Fisher 2016 B	Not performed	Not prescribed	Not prescribed
Hackett 2018	Not performed	Participants were encouraged to increase their caloric intake	Whey protein (30.8 g of protein, 0.2 g of fat, and 0.9 g of carbohydrate) 30 min after each training session
Prestes 2019	No difference in dietary intake was found between groups but data were not available	Not prescribed	Not prescribed
Walker 2016	Not performed	Not prescribed	A standardized recovery drink containing 23 g of whey protein (8.5 g leucine and 5.1 g isoleucine per 100 g), 3 g of carbohydrate, and 1.6 g of fat immediately after each training session

**Table 4:** Participants' characteristics, training status, and strength level.

Study	Participants	RT experience	Initial strength level	Training status reported in the study	Training status according to Rhea [38]
Amirthalingam 2017	Nineteen healthy males were randomly assigned to either TRAD (*n* = 9) or GVT (*n* = 10)	TRAD: 4.8 ± 4.8 years. GVT: 3.5 ± 1.0 years. More than 1 year, 3 months consistently	Not reported, but according to data, the RS on the bench press was ∼1.0	Healthy men	Untrained to highly trained

Angleri 2017	Thirty-two men (16 legs in CP, 16 in DS, and 32 in TRAD)	6.4 ± 2.0 years	Squat RS > 1.3	Well-trained young men	Recreationally to highly trained

Branderburg 2002	Eighteen university-aged male subjects: TRAD (*n* = 10); EO (*n* = 8)	At least 1 year	Bench press RS > 1.0	Trained individuals	Recreationally trained

Enes 2021	Twenty-eight healthy males: TRAD (*n* = 9); DS (*n* = 9); RP (*n* = 10)	TRAD: 4.4 ± 0.7 years DS: 5.6 ± 1.5 years RP: 5.2 ± 2.2 years. At least 2 years	RS on the squat: TRAD = 1.6 ± 0.2; DS = 1.7 ± 0.2; RP = 1.7 ± 0.2	Resistance-trained males	Recreationally trained

Fisher 2014	Forty-one participants: control^*∗*^ (*n* = 3 men and 5 women); TRAD (*n* = 4 men and 13 women); PE (*n* = 2 men and 12 women)	At least 6 months	Not reported	Trained participants	Untrained

Fisher 2016 A	Fifty-nine participants: TRAD (*n* = 10 men/9 women); EO (*n* = 10 men/10 women); SS (*n* = 10 men/10 women)	At least 6 months	Not reported	Trained participants	Untrained

Fisher 2016 B	Thirty-six subjects: TRAD (*n* = 6 men and 5 women); DS (*n* = 3 men and 8 women); HDS (*n* = 2 men and 12 women)	At least 6 months	Not reported	Trained males and females	Untrained

Hackett 2018	Twelve healthy males: TRAD (*n* = 6) and GVT (*n* = 6)	More than 1 year, 3 months consistently	Not reported, but according to data, the RS on the bench press was ∼1.0	Healthy males	Untrained to recreationally trained

Prestes 2019	Eighteen subjects (14 males and 4 females): TRAD (*n* = 9) and RP (*n* = 9)	More than 1 year	Not reported, but according to data, the RS on the bench press was ∼1.1	Trained subjects	Recreationally trained

Walker 2016	Twenty-eight men: TRAD (*n* = 10), EO (*n* = 10), and control^*∗*^ (*n* = 8)	0.5–6 years 2.6 ± 2.2 years	Not reported	Strength-trained men	Untrained to highly trained

TRAD: traditional resistance training; GVT: German volume training; RS: relative strength; EO: eccentric overload; DS: drop-set; RP: rest-pause; PE: pre-exhaustion; SS: super slow; HDS: heavy DS; ^*∗*^ = groups not considered in the calculation of standardized mean difference.

## Data Availability

All data are publicly available on original studies included.

## References

[1] Cormie P., McGuigan M. R., Newton R. U. (2011). Developing maximal neuromuscular power: Part 1--biological basis of maximal power production. *Sports Medicine*.

[2] Aagaard P. (2003). Training-induced changes in neural function. *Exercise and Sport Sciences Reviews*.

[3] Russell B., Motlagh D., Ashley W. W. (1985). Form follows function: how muscle shape is regulated by work. *Journal of Applied Physiology*.

[4] Spiering B. A., Kraemer W. J., Anderson J. M. (2008). Resistance exercise biology: manipulation of resistance exercise programme variables determines the responses of cellular and molecular signalling pathways. *Sports Medicine*.

[5] Phillips S. M. (2014). A brief review of critical processes in exercise-induced muscular hypertrophy. *Sports Medicine*.

[6] Dankel S. J., Buckner S. L., Jessee M. B. (2018). Correlations do not show cause and effect: not even for changes in muscle size and strength. *Sports Medicine*.

[7] Loenneke J. P., Dankel S. J., Bell Z. W. (2019). Is muscle growth a mechanism for increasing strength?. *Medical Hypotheses*.

[8] American College of Sports Medicine (2009). American college of sports medicine position stand. Progression models in resistance training for healthy adults. *Medicine and Science in Sports and Exercise*.

[9] Stone M. H., Plisk S. S., Stone M. E., Schilling B. K., OʼBryant H. S., Pierce K. C. (1998). Athletic performance development: volume load---1 set vs. Multiple sets, training velocity and training variation. *Strength and Conditioning Journal*.

[10] Fleck S. (1999). Periodized strength training: a critical review. *The Journal of Strength and Conditioning Research*.

[11] Kraemer W. J., Ratamess N. A. (2004). Fundamentals of resistance training: progression and exercise prescription. *Medicine and Science in Sports and Exercise*.

[12] Fleck S. J., Kraemer W. (2014). *Designing Resistance Training Programs*.

[13] Angleri V., Ugrinowitsch C., Libardi C. A. (2017). Crescent pyramid and drop-set systems do not promote greater strength gains, muscle hypertrophy, and changes on muscle architecture compared with traditional resistance training in well-trained men. *European Journal of Applied Physiology*.

[14] Schoenfeld B. (2011). The use of specialized training techniques to maximize muscle hypertrophy. *Strength and Conditioning Journal*.

[15] Angleri V., Ugrinowitsch C., Libardi C. (2020). Are resistance training systems necessary to avoid a stagnation and maximize the gains muscle strength and hypertrophy?. *Science and Sports*.

[16] Brandenburg J. E., Docherty D. (2002). The effects of accentuated eccentric loading on strength, muscle hypertrophy, and neural adaptations in trained individuals. *The Journal of Strength and Conditioning Research*.

[17] Enes A., Alves R. C., Schoenfeld B. J. (2021). Rest-pause and drop-set training elicit similar strength and hypertrophy adaptations compared with traditional sets in resistance-trained males. *Applied Physiology Nutrition and Metabolism*.

[18] Page M. J., McKenzie J. E., Bossuyt P. M. (2021). Updating guidance for reporting systematic reviews: development of the PRISMA 2020 statement. *Journal of Clinical Epidemiology*.

[19] Riva J. J., Malik K. M., Burnie S. J., Endicott A. R., Busse J. W. (2012). What is your research question? An introduction to the PICOT format for clinicians. *Journal of the Canadian Chiropractic Association*.

[20] Melibeu Bentes C., Simão R., Bunker T. (2012). Acute effects of dropsets among different resistance training methods in upper body performance. *Journal of Human Kinetics*.

[21] Amirthalingam T., Mavros Y., Wilson G. C., Clarke J. L., Mitchell L., Hackett D. A. (2017). Effects of a modified German volume training program on muscular hypertrophy and strength. *The Journal of Strength and Conditioning Research*.

[22] Gentil P., Oliveira E., de Araújo Rocha Júnior V., do Carmo J., Bottaro M. (2007). Effects of exercise order on upper-body muscle activation and exercise performance. *The Journal of Strength and Conditioning Research*.

[23] Marshall P. W., Robbins D. A., Wrightson A. W., Siegler J. C. (2012). Acute neuromuscular and fatigue responses to the rest-pause method. *Journal of Science and Medicine in Sport*.

[24] Schuenke M. D., Herman J. R., Gliders R. M. (2012). Early-phase muscular adaptations in response to slow-speed versus traditional resistance-training regimens. *European Journal of Applied Physiology*.

[25] Ouzzani M., Hammady H., Fedorowicz Z., Elmagarmid A. (2016). Rayyan-a web and mobile app for systematic reviews. *Systematic Reviews*.

[26] Higgins J. P., Altman D. G., Gøtzsche P. C. (2011). The Cochrane Collaboration’s tool for assessing risk of bias in randomised trials. *British Journal of Sports Medicine*.

[27] Higgins J. G. S. (2011). *Cochrane Handbook for Systematic Reviews of Interventions Version 5.1. 0*.

[28] Liberati A., Altman D. G., Tetzlaff J. (2009). The PRISMA statement for reporting systematic reviews and meta-analyses of studies that evaluate healthcare interventions: explanation and elaboration. *British Journal of Sports Medicine*.

[29] Higgins J. P., Thompson S. G., Deeks J. J., Altman D. G. (2003). Measuring inconsistency in meta-analyses. *British Journal of Sports Medicine*.

[30] Şahin M., Aybek E. (2019). Jamovi: an easy to use statistical software for the social scientists. *International Journal of Assessment Tools in Education*.

[31] Guyatt G., Oxman A., Vist G. (2008). GRADE: an emerging consensus on rating quality of evidence and strength of recommendations. *BMJ*.

[32] Hackett D. A., Amirthalingam T., Mitchell L., Mavros Y., Wilson G. C., Halaki M. (2018). Effects of a 12-week modified German volume training program on muscle strength and hypertrophy—a pilot study. *Sports*.

[33] Fisher J. P., Carlson L., Steele J. (2016). The effects of breakdown set resistance training on muscular performance and body composition in young men and women. *The Journal of Strength and Conditioning Research*.

[34] Fisher J. P., Carlson L., Steele J. (2016). The effects of muscle action, repetition duration, and loading strategies of a whole-body, progressive resistance training programme on muscular performance and body composition in trained males and females. *Applied Physiology Nutrition and Metabolism*.

[35] Walker S., Blazevich A. J., Haff G. G., Tufano J. J., Newton R. U., Häkkinen K. (2016). Greater strength gains after training with accentuated eccentric than traditional isoinertial loads in already strength-trained men. *Frontiers in Physiology*.

[36] Fisher J. P., Carlson L., Steele J., Smith D. (2014). The effects of pre-exhaustion, exercise order, and rest intervals in a full-body resistance training intervention. *Applied Physiology Nutrition and Metabolism*.

[37] Prestes J., A Tibana R., de Araujo Sousa E. (2019). Strength and muscular adaptations after 6 weeks of rest-pause vs. traditional multiple-sets resistance training in trained subjects. *The Journal of Strength and Conditioning Research*.

[38] Rhea M. R. (2004). Determining the magnitude of treatment effects in strength training research through the use of the effect size. *The Journal of Strength and Conditioning Research*.

[39] Martyn-St James M., Carroll S. (2006). Progressive high-intensity resistance training and bone mineral density changes among premenopausal women: evidence of discordant site-specific skeletal effects. *Sports Medicine*.

[40] Franchi M. V., Longo S., Mallinson J. (2018). Muscle thickness correlates to muscle cross-sectional area in the assessment of strength training-induced hypertrophy. *Scandinavian Journal of Medicine and Science in Sports*.

[41] Franchi M. V., Atherton P. J., Reeves N. D. (2014). Architectural, functional and molecular responses to concentric and eccentric loading in human skeletal muscle. *Acta Physiologica*.

[42] Diniz R. C. R., Tourino F. D., Lacerda L. T. (2020). Does the muscle action duration induce different regional muscle hypertrophy in matched resistance training protocols?. *The Journal of Strength and Conditioning Research*.

[43] Haun C. T., Vann C. G., Roberts B. M., Vigotsky A. D., Schoenfeld B. J., Roberts M. D. (2019). A critical evaluation of the biological construct skeletal muscle hypertrophy: size matters but so does the measurement. *Frontiers in Physiology*.

[44] Ruple B. A., Smith M. A., Osburn S. C. (1985). Comparisons between skeletal muscle imaging techniques and histology in tracking midthigh hypertrophic adaptations following 10 wk of resistance training. *Journal of Applied Physiology*.

[45] Ruple B. A., Mesquita P. H. C., Godwin J. S. (2022). Changes in vastus lateralis fibre cross-sectional area, pennation angle and fascicle length do not predict changes in muscle cross-sectional area. *Experimental Physiology*.

